# A Manual of Otology

**Published:** 1901-01

**Authors:** 


A MANUAL OF OTOLOGY. By Gorham Bacon, A. M., M. D., Professor of Otology in Cornell University Medical College, New York. With an introductory Chapter by Clarence J. Blake, M. D., Professor of Otology in the Harvard Medical School, Boston. In one handsome 12mo. volume of 422 pages, with 114 engravings and three colored plates. Cloth, $2.25, net. Lea Brothers & Co., Publishers,, Philadelphia and New York.

This is a book valuable alike to the student and practitioner. It seems to contain nothing that could be left out, and yet its completeness is far beyond what would be expected in a book of its size.

The subject of Otology is treated in a strictly scientific, yet practical manner. The necessary medicinal treatment has not been neglected, and all the important and more difficult operations are described in detail.. The new edition contains some twenty-five pages of new matter, and the number of illustrations have been considerably increased.

The book is a valuable addition to the library and is sure to receive the commendation of everyone who gets it. W. A. H.

